# Predicting Histopathological Outcomes from Cystoscopic Findings in Newly Diagnosed Bladder Cancer: A Prospective Observational Study

**DOI:** 10.7759/cureus.94251

**Published:** 2025-10-09

**Authors:** Mudasir Ahmad Tantray, Tufeel Ahmad Khan, Sajad Ahmad Malik, Sajad Ahmad Para, Saqib Mehdi, Abdul Rouf Khawaja, Arif Hamid Bhat, Saundarya Kumar Verma, Firdous Ahmad Beigh, Syed Shakeeb Arsalan

**Affiliations:** 1 Urology, Sher-i-Kashmir Institute of Medical Sciences (SKIMS), Srinagar, IND; 2 Uro-oncology, Sher-i-Kashmir Institute of Medical Sciences (SKIMS), Srinagar, IND

**Keywords:** cystoscopy, diagnostic accuracy, histopathology, muscle-invasive bladder cancer (mibc), non-muscle-invasive bladder cancer(nmibc), s: bladder cancer, transurethral resection

## Abstract

Background: Bladder cancer represents a major worldwide health challenge, with over 614,000 new diagnoses and approximately 220,000 fatalities reported globally in recent estimates, highlighting the urgent need for accurate early detection and staging to guide effective therapeutic interventions.

Objective: To assess the predictive accuracy of cystoscopic findings during initial transurethral resection of bladder tumor (TURBT) with histopathological outcomes in newly diagnosed bladder cancer, with the aim of enhancing diagnostic precision and guiding clinical management.

Methods: This prospective study enrolled 153 patients with newly diagnosed bladder tumors at a tertiary care center (January 2023-April 2025). Cystoscopic features (tumor size, number, location, morphology) were recorded using white light cystoscopy (WLC) at the time of TURBT, performed by two experienced urologic surgeons. These features were correlated with histopathological outcomes (grade, stage, subtype). Diagnostic accuracy was assessed via sensitivity, specificity, positive predictive value (PPV), and negative predictive value (NPV). Logistic regression identified predictors of histopathological outcomes, and TURBT quality was evaluated by detrusor muscle presence.

Results: Of 153 patients (male-to-female ratio: 4.88:1; mean age: 56.97 ± 15.73 years), 71.24% had non-muscle-invasive bladder cancer (NMIBC), and 28.76% had muscle-invasive bladder cancer (MIBC). Tumors sized 1-3 cm were predominant (50.33%). The prediction model achieved 81.05% accuracy, with sensitivities of 81.65% for NMIBC and 79.55% for MIBC. Low-grade Ta (LGTa) tumors had the highest prediction accuracy (83.33%). Tumor size >3 cm and sessile morphology were significant predictors of MIBC (p<0.05).

Conclusion: Initial TURBT cystoscopic findings accurately predict histopathological outcomes in newly diagnosed bladder cancer, achieving an 81.05% diagnostic accuracy. Larger (>3 cm) and sessile tumors strongly indicate MIBC, while solitary, pedunculated tumors are associated with NMIBC. The high NPV (63.64%-99.17%) supports reliable exclusion of incorrect diagnoses, particularly in resource-limited settings. High-quality TURBT, with detrusor muscle sampled in 89.4% of cases, reduces understaging and guides treatment decisions. However, lower sensitivity for high-grade NMIBC suggests that repeat TURBT could enhance staging accuracy. Future multicenter studies incorporating repeat TURBT (re-TURBT) and advanced cystoscopic technologies are needed to improve predictions and personalize treatment.

Cystoscopic findings during TURBT reliably predict histopathological outcomes, particularly for LGTa tumors, with high NPV across stages.

## Introduction

Bladder cancer stands as one of the most prevalent malignancies worldwide, contributing substantially to cancer-related morbidity and mortality. Recent global assessments indicate that in 2022 alone, more than 614,000 individuals were newly diagnosed with the disease, resulting in over 220,000 deaths, underscoring its persistent public health impact across diverse populations [1]. This heterogeneous condition is broadly categorized into non-muscle-invasive bladder cancer (NMIBC), encompassing stages Ta, T1, and carcinoma in situ (CIS), and muscle-invasive bladder cancer (MIBC), spanning stages T2 through T4. These classifications dictate markedly different treatment pathways, from conservative endoscopic resections and intravesical therapies for NMIBC to more aggressive options like radical cystectomy or systemic chemotherapy for MIBC [2, 3]. The rising incidence, influenced by factors such as aging populations, tobacco use, and environmental exposures, amplifies the necessity for precise diagnostic tools to facilitate timely and tailored patient care, especially in regions where healthcare resources are constrained [4].

Despite advancements, establishing an accurate diagnosis and stage remains complex due to the disease’s variable presentation and the potential for understaging or overstaging. White light cystoscopy (WLC) continues to serve as the primary method for initial tumor visualization and directing transurethral resection of bladder tumor (TURBT), which yields essential histopathological details on tumor grade, stage, and subtype [5]. WLC demonstrates strong sensitivity in the range of 85-90% for detecting visible lesions; however, its specificity is often compromised, leading to understaging in 20-30% of high-grade or T1 cases [6, 7]. Emerging adjunctive modalities, including narrow-band imaging (NBI) and blue light cystoscopy (BLC), have shown promise in enhancing the identification of flat or subtle lesions like CIS, yet their adoption is limited by cost, availability, and the need for specialized equipment [8, 9]. Furthermore, the effectiveness of TURBT hinges on procedural quality, particularly the sampling of detrusor muscle, as its absence can elevate understaging risks to as high as 50% in T1 tumors, potentially delaying appropriate interventions [10].

In light of these challenges, this prospective study explores the association between intraoperative cystoscopic observations during TURBT and subsequent histopathological results in a cohort of 153 patients presenting with primary bladder tumors. By analyzing key cystoscopic attributes such as tumor dimensions, multiplicity, site, and shape, the investigation aims to quantify their predictive utility, appraise TURBT procedural standards, and discuss broader applications for improving bladder cancer care across varied healthcare environments.

## Materials and methods

Study design

This prospective observational study was conducted at a tertiary care center from January 2023 to April 2025. Of 171 consecutive patients with suspected bladder tumors, 153 were included after meeting eligibility criteria. Eighteen patients were excluded due to the absence of detrusor muscle in TURBT specimens, ensuring diagnostic reliability. The study adhered to the 1964 Helsinki Declaration and its amendments, with ethical approval from the Institutional Ethical Committee, SKIMS Srinagar. Written informed consent was obtained from all participants.

Sample size calculation

The sample size was calculated to detect a clinically meaningful correlation between cystoscopic findings and histopathological outcomes, with an expected sensitivity of 80% for predicting NMIBC. Assuming a 5% margin of error, 80% power, and a 10% dropout rate, a minimum of 150 patients was required. The final sample of 153 patients was deemed sufficient to achieve statistical reliability.

Inclusion criteria

Patients with suspected bladder tumors identified on initial cystoscopy, confirmed by imaging (e.g., CT urography, ultrasound) or clinical presentation (e.g., hematuria), and planned for TURBT.

Exclusion criteria

Patients with prior bladder cancer treatment, recurrent tumors, absent detrusor muscle in TURBT specimens, or tumors other than urothelial BC, like lymphoma, adenocarcinoma, were excluded from the study.

Data collection

Cystoscopic data: During TURBT, tumor characteristics were systematically recorded using WLC by two experienced urologists with over 10 years of expertise to minimize interobserver variability. Parameters included tumor size (<1cm, 1-3cm, >3cm), number (single or multiple), location (bladder neck, trigone, lateral walls, posterior wall, anterior wall, dome), and morphology (papillary, sessile, flat). A standardized checklist was used to ensure consistency, and discrepancies were resolved through post-procedure consensus meetings. To minimize interobserver variability, cystoscopic findings were recorded independently by two experienced urologists in a random subset of 50 cases (32.7% of total), followed by consensus resolution. Agreement was quantified using Cohen’s kappa (κ). For tumor size categorization, κ=0.88 (95% confidence interval (CI): 0.79-0.97, almost perfect agreement). For morphology (pedunculated vs. sessile), κ=0.82 (95% CI: 0.71-0.93, substantial agreement). For number (solitary vs. multiple), κ=0.91 (95% CI: 0.83-0.99). These values exceed recommended thresholds (κ>0.70) for reliable classification, supporting reproducibility.

Histopathological data

TURBT specimens were evaluated by a uropathologist for tumor grade (low or high, WHO 2016 classification), stage (Ta, T1, T2, CIS, TNM system), histological subtype, and detrusor muscle presence. For tumors staged beyond T2, histopathological assessment relied on TURBT specimens with deep resection to include muscularis propria, supplemented by imaging (CT/MRI) for clinical staging when necessary, as TURBT alone is limited for T3/T4 staging.

Clinical Data

Patient demographics (age, sex), smoking history (pack-years), and comorbidities (e.g., diabetes, hypertension) were collected via structured questionnaires and stored in a secure digital database compliant with data protection standards.

Methodology

Recruitment and Consent

Eligible patients were identified during cystoscopy performed as part of the TURBT, informed of study objectives, and provided written consent following detailed counseling. Consent forms were standardized to ensure clarity and compliance with ethical guidelines.

TURBT Protocol

TURBT was performed under general or spinal anesthesia using a standardized technique to achieve complete tumor resection and detrusor muscle sampling by a team of multiple experienced urologists at our tertiary care center. To ensure consistency and minimize interobserver variability, cystoscopic findings were independently recorded by two experienced urologists during each procedure. A checklist documented intraoperative findings, with video recordings reviewed for quality control. Bipolar or monopolar resection was used based on the surgeon’s preference, with equipment calibrated per the manufacturer's guidelines. No major deviations from the protocol occurred; minor variations (e.g., anesthesia type or resection method) were documented and had no significant impact on outcomes. No major deviations from the protocol occurred; minor variations (e.g., anesthesia type or resection method) were documented and had no significant impact on outcomes.

Quality Assessment

Detrusor muscle presence in TURBT specimens was a predefined quality metric. Understaging rates were evaluated by comparing initial TURBT staging with subsequent histopathological findings, using chi-square tests to assess significance.

Statistical analysis

Data were analyzed using IBM SPSS Statistics (version 27.0). Descriptive statistics (means, standard deviations, frequencies, percentages) summarized patient demographics, cystoscopic findings, and histopathological outcomes. Multivariable logistic regression models identified predictors of histopathological outcomes (e.g., MIBC or high-grade tumors), adjusting for confounders such as age, sex, and smoking status. Variables for regression were selected based on univariate analysis (p<0.10) and clinical relevance, with crude and adjusted odds ratios (ORs) reported with 95% confidence intervals (CIs). A p-value <0.05 was considered statistically significant. Diagnostic accuracy was assessed via sensitivity, specificity, positive predictive value (PPV), and negative predictive value (NPV), with 95% CIs. Receiver operating characteristic (ROC) curve analysis was performed to evaluate the model’s discriminative ability (area under the curve (AUC) reported). Interobserver agreement was quantified using Cohen’s kappa. Missing data (<5% of cases) were handled using listwise deletion, with sensitivity analyses to assess potential bias. Interim analyses at 6-month intervals ensured data integrity, with no major protocol deviations noted.

## Results

Demographic profile

The study included 153 patients (127 males, 26 females; male-to-female ratio: 4.88:1; mean age: 56.97 ± 15.73 years), as shown in Table 1. The predominant age group was 50-79 years (71.24%, 109/153), followed by 30-49 years (17.65%, 27/153), ≤29 years (5.23%, 8/153), and ≥80 years (5.86%, 9/153), as presented in Table 2.

**Table 1:** Age and gender profile

Variable	Overall	Male	Female
Number (n)	153	127	26
Mean age(years)	56.97 (+-15.73)	56.92 (+-16.22)	57.20 (+-13.45)
Range(years)	18-85	18-85	30-75
Male: female ratio	4.88:1		

**Table 2:** Gender distribution across age groups

Age group(years)	Males(n)/%	Females(n)/%	Total(n)/%
<=29	8 (5.23)	0 (0.00)	8 (5.23)
30-49	22 (14.38)	5 (3.27)	27 (17.65)
50-79	90 (58.82)	19 (12.42)	109 (71.24)
>=80	9 (5.86)	0.00 (0.00)	9 (5.86)

Tumor characteristics on white light cystoscopy

Cystoscopic evaluation revealed a notable distribution of tumor sizes among 153 patients, with 1-3 cm tumors being most prevalent (50.33%), followed by >3 cm tumors (30.07%) and <1 cm tumors (19.60%). Pedunculated tumors dominated across all sizes (86.7% for <1 cm, 63.6% for 1-3 cm, 65.2% for >3 cm), while sessile tumors became more prevalent with increasing size. Notably, all <1 cm tumors were solitary, whereas multiple tumors emerged in larger size groups (40.26% for 1-3 cm, 45.65% for >3 cm), as detailed in Table 3. Tumor locations underscored a clear pattern: the lateral walls accounted for the majority of tumors (57.51%, 88/153), followed by the posterior wall (26.14%, 40/153), trigone (7.84%, 12/153), dome (3.92%, 6/153), anterior wall (3.26%, 5/153), and bladder neck (1.30%, 2/153), as presented in Table 4.

**Table 3:** Distribution of tumor characteristics by size on cystoscopy

Variables	<1 cm (n=30, 19.60%)	1-3 cm (n=77, 50.33%)	>3 cm (46, 30.07)
Pedunculated tumors	26 (86.67)	49 (63.64)	30 (65.22)
Sessile tumors	4 (13.33)	28 (36.36)	16 (34.78)
Solitary bladder tumors	30 (100)	46 (59.74)	25 (54.35)
Multiple bladder tumors	Nil	31 (40.26)	21 (45.65)

**Table 4:** Tumor location on cystoscopy

Location	Number (n)	Percentage (%)
Lateral walls	88	57.51
Posterior wall	40	26.14
Anterior wall	5	3.26
Dome	6	3.92
Trigone	12	7.84
Bladder neck	2	1.3

Histopathological outcomes

NMIBC was diagnosed in 109 patients (71.24%), and MIBC in 44 (28.76%). Among tumor stages, low-grade Ta (LGTa) tumors were most common (43.14%, 66/153), followed by pT2 (28.76%, 44/153), high-grade pT1 (HG pT1, 13.07%, 20/153), high-grade Ta (HGTa, 11.76%, 18/153), and low-grade pT1 (LG pT1, 3.27%, 5/153). Prediction accuracy for these categories is detailed in Table 5.

**Table 5:** Prediction accuracy of NMIBC and MIBC by grade and stage NMIBC: muscle-invasive bladder cancer; LGTa: low-grade Ta; HGTa: high-grade Ta; PT2: muscularis propria

Final Histopathology	Confirmed (n)%	Correctly Predicted (n) %
NMIBC	109 (71.24)	89 (81.65)
MIBC	44 (28.76)	35 (79.55)
LGTa	66 (43.14)	57 (86.36)
HGTa	18 (11.76)	13 (72.22)
LG pT1	5 (3.27)	4 (80)
HG pT1	20 (13.07)	15 (75)
PT2	44 (28.76)	35 (79.55)

Diagnostic accuracy

The prediction model, developed to correlate cystoscopic findings with histopathological outcomes, demonstrated an overall accuracy of 81.05% (124/153 cases correctly identified; 95% CI, 74.26%-86.43%).ROC curve analysis yielded an AUC of 0.84 (95% CI, 0.78-0.89) for the overall model, indicating good discriminative ability for distinguishing between NMIBC and MIBC.

For NMIBC, comprising 109 patients, the model correctly predicted 89 cases, yielding a sensitivity of 81.65% (95% CI, 73.43%-87.77%), specificity of 79.55% (95% CI, 65.51%-88.98%), PPV of 90.82% (95% CI, 83.49%-95.09%), NPV of 63.64% (95% CI, 50.39%-75.15%) and an AUC of 0.83 (95% CI, 0.77-0.88).

For MIBC, encompassing 44 patients, the model accurately identified 35 cases, with a sensitivity of 79.55% (95% CI, 65.51%-88.98%), specificity of 81.65% (95% CI, 73.43%-87.77%), PPV of 63.64% (95% CI, 50.39%-75.15%), NPV of 90.82% (95% CI, 83.49%-95.09%) and an AUC of 0.82 (95% CI, 0.75-0.87).

Stage-specific diagnostic performance was assessed for each histopathological category. For low-grade Ta (LGTa) tumors, which included 66 patients, the model correctly predicted 57 cases, achieving a sensitivity of 86.36% (95% CI, 76.01%-92.79%), specificity of 79.31% (95% CI, 68.93%-87.09%), PPV of 77.03% (95% CI, 66.25%-85.35%), NPV of 87.88% (95% CI, 78.07%-93.81%), and an AUC of 0.86 (95% CI, 0.80-0.91). For high-grade Ta (HGTa) tumors, comprising 18 patients, 13 cases were accurately predicted, resulting in a sensitivity of 72.22% (95% CI, 49.13%-87.50%), specificity of 82.22%, PPV of 35.14%, NPV of 95.69%, and an AUC of 0.78 (95% CI, 0.69-0.85). For LG pT1 tumors, with only five patients, four cases were correctly identified, yielding a sensitivity of 80.00% (95% CI, 37.55%-96.38%), specificity of 81.08%, PPV of 12.50%, NPV of 99.17%, and an AUC of 0.81 (95% CI, 0.67-0.90). For HG pT1 tumors, involving 20 patients, the model correctly predicted 15 cases, with a sensitivity of 75.00% (95% CI, 53.13%-88.80%), specificity of 81.95%, PPV of 38.46%, NPV of 95.61%, and an AUC of 0.79 (95% CI, 0.71-0.86). For pT2 tumors, encompassing 44 patients, 35 cases were accurately predicted, demonstrating a sensitivity of 79.55% (95% CI, 65.51%-88.98%), specificity of 81.65%, PPV of 63.64%, NPV of 90.82%, and an AUC of 0.82 (95% CI, 0.75-0.87).

Univariable logistic regression analyses identified strong associations between specific cystoscopic features and MIBC. Tumors larger than 3 cm were associated with a higher likelihood of MIBC (crude OR = 8.85, 95% CI: 3.97-19.74, p < 0.001), as was sessile morphology (crude OR = 6.66, 95% CI: 2.94-15.18, p < 0.001). In contrast, the presence of multiple tumors showed a weaker, non-significant association with MIBC (crude OR = 1.53, 95% CI: 0.74-3.19, p = 0.24).

Multivariable logistic regression analysis identified tumor size greater than 3 cm as a significant predictor of MIBC, with an OR of 2.8 (95% CI, 1.4-5.6; p=0.003). Similarly, sessile tumor morphology was a significant predictor, with an OR of 3.1 (95% CI, 1.6-6.0; p=0.001). The presence of detrusor muscle, a critical indicator of TURBT quality, was confirmed in 89.4% of specimens. Analysis revealed that understaging rates were significantly higher in specimens lacking detrusor muscle, with a statistically significant association (p=0.02).

## Discussion

This study demonstrates a robust correlation between cystoscopic findings during TURBT and histopathological outcomes, achieving an overall accuracy of 81.05%, comparable to advanced imaging modalities like multiparametric MRI (75-85%) [11]. The high sensitivity for LGTa tumors (86.36%) underscores the model’s effectiveness in identifying NMIBC, critical for guiding conservative treatments such as intravesical therapy [2]. Lower sensitivities for HGTa (72.22%) and HG pT1 (75.00%) indicate challenges in predicting high-grade lesions, possibly due to molecular heterogeneity or overlapping cystoscopic features with MIBC [12]. The high NPV across stages (63.64%-99.17%) supports reliable exclusion of incorrect diagnoses, minimizing overtreatment in NMIBC or undertreatment in MIBC [7].

The predominance of 1-3 cm, pedunculated, solitary tumors aligns with the high prevalence of LGTa tumors (43.14%), which are typically less aggressive [3]. Larger tumors (>3 cm) and sessile morphology were associated with HG pT1 and pT2 tumors, reflecting their higher risk of progression [6]. The low incidence of bladder neck tumors (1.30%) suggests minimal obstruction at diagnosis, potentially influencing clinical presentation [8].

TURBT quality was high, with detrusor muscle present in 89.4% of specimens, reducing understaging risks compared to studies reporting 50% understaging without detrusor muscle [10]. The reliance on WLC, standard in many clinical settings, limits CIS detection compared to NBI or BLC, highlighting the need for broader access to advanced technologies [9]. Tumor size >3 cm and sessile morphology as predictors of MIBC provide prognostic insights, guiding decisions on radical cystectomy or bladder-sparing therapies [2, 3].

The study focused exclusively on initial TURBT findings to evaluate immediate cystoscopic-histopathological correlations in newly diagnosed bladder cancer, and thus, findings from repeat TURBT (re-TURBT) were not included. Re-TURBT is recommended by guidelines, particularly for high-risk NMIBC such as high-grade T1 tumors, to confirm complete resection and detect upstaging, which occurs in 20-30% of T1 cases [5, 7]. By design, our study prioritized the initial diagnostic procedure to establish baseline correlations, as routine re-TURBT was performed only for select high-risk cases per clinical protocol, introducing variability that could confound our primary objective. However, this focus limits our ability to assess upstaging or residual disease, which are critical for long-term management. We recommend that future studies incorporate re-TURBT findings to quantify upstaging rates and validate the prognostic utility of initial cystoscopic observations in NMIBC, particularly for high-grade lesions where understaging risks are highest. Additionally, as a single-center study conducted at a tertiary care facility, the findings may lack generalizability to diverse healthcare settings with varying resources and expertise.

## Conclusions

Cystoscopic observations during initial TURBT effectively predict histopathological outcomes in newly diagnosed bladder cancer, with an overall diagnostic accuracy of 81.05%. Larger tumors (>3 cm) and sessile morphology strongly indicate MIBC, whereas solitary, pedunculated tumors are closely linked to NMIBC. The model’s high NPV, ranging from 63.64% to 99.17% enhances its ability to exclude incorrect diagnoses, especially in settings with limited access to advanced imaging or cystoscopic technologies like NBI or BLC. These results highlight the critical role of high-quality TURBT, with detrusor muscle sampled in 89.4% of cases, in reducing understaging and informing treatment choices, such as bladder-sparing approaches versus early radical cystectomy. However, the lower sensitivity for detecting high-grade NMIBC (e.g., HGTa and HG pT1) suggests that reTURBT could improve staging precision, particularly for high-risk cases, by identifying potential upstaging. Future studies should include reTURBT data and investigate advanced cystoscopic methods in larger, multicenter cohorts to improve predictions for high-grade NMIBC and support tailored treatment plans.
